# Linking Foraging Decisions to Residential Yard Bird Composition

**DOI:** 10.1371/journal.pone.0043497

**Published:** 2012-08-22

**Authors:** Susannah B. Lerman, Paige S. Warren, Hilary Gan, Eyal Shochat

**Affiliations:** 1 The Graduate Program in Organismic and Evolutionary Biology, University of Massachusetts – Amherst, Amherst, Massachusetts, United States of America; 2 The Department of Environmental Conservation, University of Massachusetts – Amherst, Amherst, Massachusetts, United States of America; 3 The Global Institute of Sustainability, Arizona State University, Tempe, Arizona, United States of America; 4 Yeruham Center of Ornithology and Ecology, Yeruham, Israel; University of Western Ontario, Canada

## Abstract

Urban bird communities have higher densities but lower diversity compared with wildlands. However, recent studies show that residential urban yards with native plantings have higher native bird diversity compared with yards with exotic vegetation. Here we tested whether landscape designs also affect bird foraging behavior. We estimated foraging decisions by measuring the giving-up densities (GUD; amount of food resources remaining when the final forager quits foraging on an artificial food patch, i.e seed trays) in residential yards in Phoenix, AZ, USA. We assessed how two yard designs (mesic: lush, exotic vegetation; xeric: drought-tolerant and native vegetation) differed in foraging costs. Further, we developed a statistical model to calculate GUDs for every species visiting the seed tray. Birds foraging in mesic yards depleted seed trays to a lower level (i.e. had lower GUDs) compared to birds foraging in xeric yards. After accounting for bird densities, the lower GUDs in mesic yards appeared largely driven by invasive and synanthropic species. Furthermore, behavioral responses of individual species were affected by yard design. Species visiting trays in both yard designs had lower GUDs in mesic yards. Differences in resource abundance (i.e., alternative resources more abundant and of higher quality in xeric yards) contributed to our results, while predation costs associated with foraging did not. By enhancing the GUD, a common method for assessing the costs associated with foraging, our statistical model provided insights into how individual species and bird densities influenced the GUD. These differences we found in foraging behavior were indicative of differences in habitat quality, and thus our study lends additional support for native landscapes to help reverse the loss of urban bird diversity.

## Introduction

Urbanization alters the composition and function of landscapes, rendering certain areas unsuitable for native wildlife [1]. Patterns emerging from urban studies indicate that bird species richness and evenness decline while biomass and density increase [2]–[5]. Recently, studies have shown that within urban areas, landscape designs that mimic the wildlands being replaced (i.e. more native-like) are more diverse compared with landscapes with exotic vegetation (i.e. more urban [6]–[8]). These and other studies highlight ways to reconcile human development with ecosystem function [9].

Understanding the processes that lead to changes in community composition and structure requires more detailed studies of individuals and populations. Shochat et al. [10] compared individual foraging behavior between urban and desert bird communities in Phoenix, Arizona. They quantified the quitting point of foraging by measuring the giving-up densities (GUD: the density of resources remaining after foraging stops; [11]) for the final forager visiting artificial food patches. This study provided an excellent reference for comparing urban and wildland habitats in terms of productivity, structure and function. They found that urban birds exhibited lower GUDs (consumed more resources) than desert birds.

When testing how competition influenced foraging decisions, Shochat et al. [10] compared foraging decisions between two different urban landscape designs (mesic: yards landscaped with exotic plants, more urban-like; xeric: yards landscaped with native plants, more desert-like), and found lower GUDs in mesic yards compared to xeric yards. These preliminary results had too low a sample size to be conclusive, but suggested that differences in mesic and xeric landscapes may support a bird community structure similar to that found at a broader scale between urban and desert habitats, respectively. In addition to the small sample size, the Shochat et al. [10] study lacked definitive information on the density of foragers and species identity. Furthermore, their index of efficiency did not accurately measure the rate of seed consumption for each species, weakening the inference that highly efficient foragers dominate urban bird communities. Lerman and Warren [8] found that xeric yards in Phoenix, AZ were mini refugia for native birds, and many birds from the Sonoran desert bird community were represented. However, it is unclear how urban features such as elevated resources from human subsidies (e.g. exotic vegetation, refuse and bird feeders; [12], [13]) influence the bird community in xeric yards. Furthermore, a number of studies have shown that for adult birds and squirrels, predation pressure in urban settings is relaxed [10], [14]–[16]. These distinct urban features might lead to different foraging decisions by birds in xeric yards than in native desert areas, despite the structural similarities of these two habitat types.

### Optimal Foraging Theory and Giving Up Densities

Optimal foraging theory provides a useful framework for testing how animals perceive habitat quality [17], [18]. An animal behaving optimally quits foraging a patch when the marginal profits (i.e. energy gains) equal the marginal costs of foraging (e.g. predation risk, metabolic cost, and missed opportunity costs; [11]). Using artificial and depletable food patches, we can manipulate resource-consumer relationships, measure foraging decisions, and highlight some of the costs associated with foraging. Specifically, as food becomes depleted, the forager experiences diminishing returns; finding additional food items becomes increasingly difficult and the benefits of foraging in the patch no longer outweigh the costs [17]. The quitting point or GUD provides a quantitative assessment on a forager’s perception of foraging costs within the environment while also providing a measurement for efficiency of the final forager [11], [17]. A forager will quit a patch earlier in higher quality environments due to greater abundances of alternative food resources [19]–[21].

The GUD method hails from an established conceptual framework [11], is simple to execute, and yet is robust in its ability to link energetic gain from foraging to urban bird community structure. One of the strengths of the method includes the ability of the foragers to remain in their natural environment with their natural competitors, resources, and alternative activities. Many GUD studies have focused on birds due to their ability to exploit novel food patches (e.g., bird feeders), and their mobility [11], [22], [23].

Here we build upon the work of Shochat et al. [10] by more rigorously testing the differences in foraging behavior between mesic and xeric landscape designs within the city, and identifying some potential mechanisms for these differences in bird composition. Understanding how bird behavior varies across the urban landscape may provide insights on whether native landscaping in the city can support the same functions as native desert environments. For our study, we tested whether the costs associated with foraging differed between the mesic and xeric yards. We used seed trays as artificial food patches, and measured the amount of seed left in the tray after 24 hours. This measurement represented the GUD of the final forager visiting the tray. The logic is that the last species to visit a tray was able to locate food items after other species quit and that the food remaining on the tray represents the foraging decision of this final forager [11]. In our study, we advanced the GUD method to disentangle some of the effects of the bird community composition, the density of competitors and behavioral differences, and how these factors affected foraging decisions between two different landscape designs. If xeric yards function more like the desert in terms of resource availability and foraging costs than the urban environment (mesic yards), then we expected to see higher GUDs in xeric than mesic yards. If however, the altered resources and predation risks of the urban environment yield similar costs and benefits of foraging in xeric and mesic yards, we expected to see low GUDs in both yard types.

## Materials and Methods

### Study Area

Nestled in the northern limits of the Sonoran desert, Phoenix, AZ is one of the fastest-growing cities in the US. We conducted our experiments in 20 residential yards located in the greater Phoenix metropolitan area, ten of which were landscaped with a mesic design (‘more urban’ - turf ground cover and exotic vegetation) and ten of which were of xeric design (‘more desert’ - crushed gravel with drought-tolerant and/or native vegetation). All 20 yards were independent from each other; there was a minimum distance of 3 km between yards. Therefore it was unlikely that an individual foraged in more than one of the 20 focal yards. All experimental setups were located within 20 m of a residence. All homeowners removed bird feeders before and during the experiments. We calculated the distance to large desert patches from the 20 residential yards to ensure proximity did not confound results regarding the bird community. We found no significant difference between the mesic and xeric yard proximity to desert patches (ANOVA; F = 0.59, df = 1,19, P = 0.45); mean distance between mesic yards and desert patches was 5.99 km (±2.37 SD) and mean distance for xeric yards was 4.98 km (±3.39 SD). Experiments were conducted between February and April 2010. Temperatures were relatively stable; with a mean minimum temperature of 11.64°C (±2.42 SD) and maximum temperature of 24.57°C (±4.19 SD). Within Phoenix residential areas, the design types of individual yards generally mimic those of their neighbors [8] suggesting that similar foraging opportunities existed within close proximity of the focal yard. Housing density and lot size within Phoenix, AZ are also relatively homogenous [24], suggesting the amount of available habitat within close proximity of the focal residential yards did not vary.

### Seed Trays

We mixed 20 g of millet seed with 3 kg of sifted sand in green plastic trays (36-cm diameter) to simulate resource patches. Depth of sand in the trays was approximately 6.5 cm. Sites were baited with identical seed trays at least 24 h in advance, or as long as necessary to detect visitation. We placed two trays in each yard, and each tray was placed on a stool (0.7 m tall) for a 24 h trial. After 24 h, each tray was removed from the site and brought back to the lab for processing. We sifted the trays by pouring the sand and seed mixture through a sifting screen twice. The remaining seeds were separated from the empty hulls and any debris, and then weighed to calculate the GUD to the nearest 0.01 g.

### Assessing the Perceived Risk of Predation

Perceived risk of predation influences foraging decisions: in general, foragers in risky patches quit foraging earlier and at higher resource harvest rates than foragers in safe patches [10], [11], [15], [25]. Distance from cover has been shown to correlate positively with perceived predation risk in habitats in which aerial predators represented the primary threat [11], [26], [27], [28]. Alternatively, cover may hide predators such as cats, and distance from cover may correlate negatively with perceived predation risk. Here, we tested whether distance from cover influenced foraging efficiency. We placed one tray next to large bushes and shrubs and one tray three meters away from vegetation. If cats or other ground predators posed a predation risk and used bushes for shelter, then we expected the birds to deplete more resources in the trays away from bushes (i.e. higher GUDs from the trays next to bushes). If aerial predators posed a predation risk, then we expected the birds to deplete more resources in the trays close to bushes (i.e. higher GUDs from the open trays).

We assumed that an individual forager’s choice to quit foraging at a tray reflected the optimal decision for the relative amounts of risk and of food available in the two microhabitats at that time. While foragers may not have ideal knowledge of food availability in the two microhabitats, it was possible that foragers assessed whether a tray had been visited previously and how vigorously it had been searched for food. At the beginning of each trial, the sand in the trays was smooth and some seed was visible. Some indications of prior visitation included footprints, uneven sand surface, and no visible seed. Concurrent measures of GUDs in the two microhabitats were necessary to avoid confounding daily variation with differences in use of the microhabitats.

### Video Recording and Analysis

We used Trendnet TV-IP110 network IP cameras with Active Webcam video recording software (PY Software, version 11.5) on Lenovo G550 Laptop Computers to record foraging behavior for the entire 24 h experiments for all trays. Cameras were housed in plexiglass cases, secured to the stools and placed approximately 0.75 cm from the seed tray. To facilitate data file management, we divided the 24 h recording time into two 12 h files. Frame rates were set at 20 fps, with a maximum file size of 1100 MB. Files were in WMV format, and optimized to automatically continue recording in a new file when the maximum file size was reached.

We used JWatcher (http://www.jwatcher.ucla.edu/) version 1.0 with VLC media play to score foraging behaviors from the videos. We focused on four events from the videos: species identification for each tray, the total number of foraging pecks for each species per tray, time spent foraging on the tray, and the minimum number of individuals visiting each tray. To calculate the second two events, we logged every peck per species per tray. We then calculated the total amount of time each species spent foraging on each tray. Because the birds in our study were not marked, we could only calculate the minimum number of individuals per tray as an estimate of density. For example, when a tray had three female house sparrows (*Passer domesticus*) on the tray at once, and then later, had two male and one female house sparrows, we recorded a minimum of five individuals, assuming that the female was a repeat visitor. Counting the pecks of each individual forager allowed us to calculate the total number of pecks for each tray, the total number of pecks for each species per tray, and the cumulative number of pecks per tray at the time when each species quit foraging on each tray. Video monitoring also allowed us to confirm that only birds visited the seed trays.

### Statistical Analysis

We used JMP 8 statistical software for all analyses except when noted otherwise, and set the significance level at P = 0.05. We checked for normality of the residuals of the data and transformed when necessary.

### The GUD Peck Model

One of the limitations of the GUD method is that it only measures the foraging decision of the last species visiting the artificial seed trays [11]. As typically used, the method does not provide information about the decisions of other species foraging on the tray prior to the last species. In addition, the method lacks the capacity to assess the true effort expended by the forager and the actual resources consumed, and thus the GUD only provides a rather imprecise index of efficiency. To address these limitations, we developed an experimental and analytical method for estimating the GUD for all species visiting each seed tray by calculating a GUD-to-peck relationship to increase the amount of data obtained from a single seed tray.

When birds forage, they exhibit a highly stereotyped pecking motion. This provides an avenue for estimating foraging effort on the trays through video monitoring. We hypothesized that the number of pecks an individual engages in during a visit to the tray would be proportional to the amount of seed consumed. If so, then we could estimate the GUD for each visitor from knowing the final GUD on the tray and the number of pecks each visitor engaged in.

**Figure 1:**
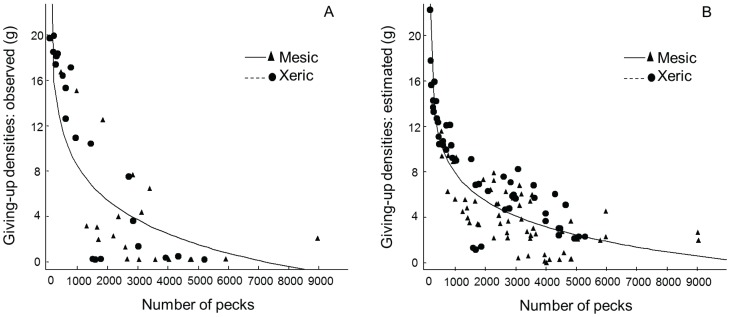
GUD-peck Model. Simplified GUD-peck model based on the final forager, n = 42 (a) to estimate GUDs for all foragers, n = 114 (b) visiting the seed trays. Final model included density of foragers visiting the trays and species identity, and had a strong fit (r^2^ = 0.83). To test the accuracy of this model, we compared the observed GUD values against the predicted GUD values based on the model (Pearson Correlation = 0.91). Estimated GUDs were based on the cumulative number of pecks on the seed tray when each species quit foraging. Triangles are GUDs from mesic yards and Squares are GUDs from xeric yards.

First, we tested whether the final GUD was proportional to the total number of pecks on a tray. We used video monitoring to count the number of pecks made by each visitor for all trays in the 20 yards (n = 40). For two of the twenty yards, we conducted an additional foraging trial because one tray in each of these two yards was not visited during the first trial. We measured the GUD (amount of seed remaining in the tray when the final forager quit) for every tray that birds visited for each trial (n = 42 individuals). Figure 1a shows the approximately linear relationship between GUD and pecks.

We then developed a GUD-Peck model to estimate the GUD for all other foragers aside from the final forager (n = 72 individuals) visiting the 42 trays (Fig. 1b). We incorporated the effects of species identity and number of visitors to the tray to account for potential factors influencing the GUD. This model assumes that all pecks were equally likely to yield food, all pecks yielded the same amount of food, and all species acquired about the same amount of food per peck. To test the accuracy of the model, we compared the observed GUD values against the predicted GUD values based on the model (Pearson Correlation = 0.91). We then used the model to estimate the GUD for the remaining 72 observations of individual birds based on the cumulative number of pecks observed by the time that each species quit foraging at each tray. Specifically, by estimating the GUD for all individual visitors, we used the estimated GUD of the last visitor of a particular species as the GUD for that species for each tray. For example, when species X quit foraging at a tray, our number included pecks by all species that had visited the tray previously, but no pecks by any later visitors to that tray.

### Calculating Rate of Consumption

We used the GUD-Peck model to calculate the rate of seed consumption for each species visiting the trays by entering the total number of pecks per species into the GUD- Peck model. We then calculated the total amount of time each species spent pecking at each tray by summing all foraging bouts for each species. A foraging bout began with the species’ first peck and ended when at least one minute passed without a peck for the focal species. Since we did not include the time period prior to the first peck (i.e. time between arrival on tray and first peck) or the time period after the last peck and departure from the tray, our calculations represent the minimum residency time and likely underestimated the actual residency time. However, we sub sampled 5% of the foraging bouts to estimate the amount of time spent sitting on the tray prior and after pecking and found this time to be minimal. Birds began pecking within five seconds upon arrival, and departed the tray within ten seconds of the final peck. The GUD-Peck model performed poorly for instances in which a visitor to the tray had very few pecks (<40). Here, the model estimated a negative number for seed consumed for these cases, and therefore we omitted these data to better estimate the efficiency.

### Perceived Predation Risk

The shrub species, density and configuration significantly differed between mesic and xeric yards [8]. Furthermore, the vegetation variation might provide different refuges from predators and also impact a bird’s capacity to detect predators [29]. To test whether distance from cover influenced foraging decisions, we conducted two separate paired-samples t-tests: one for all the species foraging in ten mesic yards and one for all the species foraging in the ten xeric yards. We compared the GUD (response variable) for each species for each yard, from two different conditions: trays out in the open (3 m away from vegetation) and trays close to bushes. The number of species visiting each tray ranged between one and six. For the 10 mesic yards, there were 26 paired observations and for the 10 xeric yards, there were 19 paired observations. These pairs only included a species within each yard that visited both trays. We cannot be certain whether the lack of visitation was an active decision. Therefore, we conducted an additional paired-samples t-test for the 34 paired observations from the mesic yards and the 27 paired observations for xeric yards (which included the trays not visited, and hence the GUD was 20 g) and obtained similar results.

### Bird Community

We tested whether the species visiting the seed trays differed between the two yard types using a Multi-response Permutation Procedure (MRPP). This nonparametric procedure tested the null hypothesis that the two groups (mesic and xeric yards) did not differ. MRPP compared the observed intra-group average distance with the average distance expected for all other combinations under the assumption of the null hypothesis [30]. We included minimum number of individuals per species per yard as the density estimate. The coefficient of variation for the data was high (>100) so we conducted a row normalize standardization procedure to calculate the species composition within each yard [30]. We dropped species occurring at less than 5% of the sites since they were not sampled sufficiently and the data could not reliably characterize their ecological patterns [30]. Our data had many zeroes (>30% per site) and therefore we used the Bray Curtis distance measurement. Results were based on 999 permutations. We also performed an analysis of group similarities (ANOSIM) and obtained similar results. We conducted these analyses using R [31] (2008) with the Vegan package [32].

### Landscape Design Differences

To test whether foraging decisions differed between the two yard types, we pooled the GUDs for all species for each yard type and then conducted an analysis of variance (ANOVA). The independent variable was yard type, and the response variable was the GUD. Using the pooled data, we then conducted an analysis of covariance (ANCOVA) with GUD as the response, yard type as the factor, and bird density (minimum number of individuals visiting a tray) as a covariate to address how bird densities might decrease the GUD. We also tested whether individual species altered their foraging behavior between the two different yard types. We focused on the four species that had multiple observations within mesic and xeric yards (i.e. visited both the mesic and xeric yards; Abert’s towhee [*Pipilo aberti*], curve-billed thrasher [*Toxostoma curvirostre*], house finch [*Carpodacus mexicanus*], and house sparrow). Here we averaged the GUD for each species for each yard type. Since sample sizes were small, we used a nonparametric approach (Wilcoxon Test); GUD was the response variable and yard type was the independent variable. In addition, we compared the foraging efficiency (as calculated by our model; the response variable) among these four species (independent variable) for each yard design using the Kruskal-Wallis Test.

### Ethics Statement

The protocol was approved by the Animal Care Office of the University of Massachusetts (IACUC Permit Number: 2009–0007).

## Results

**Figure 2:**
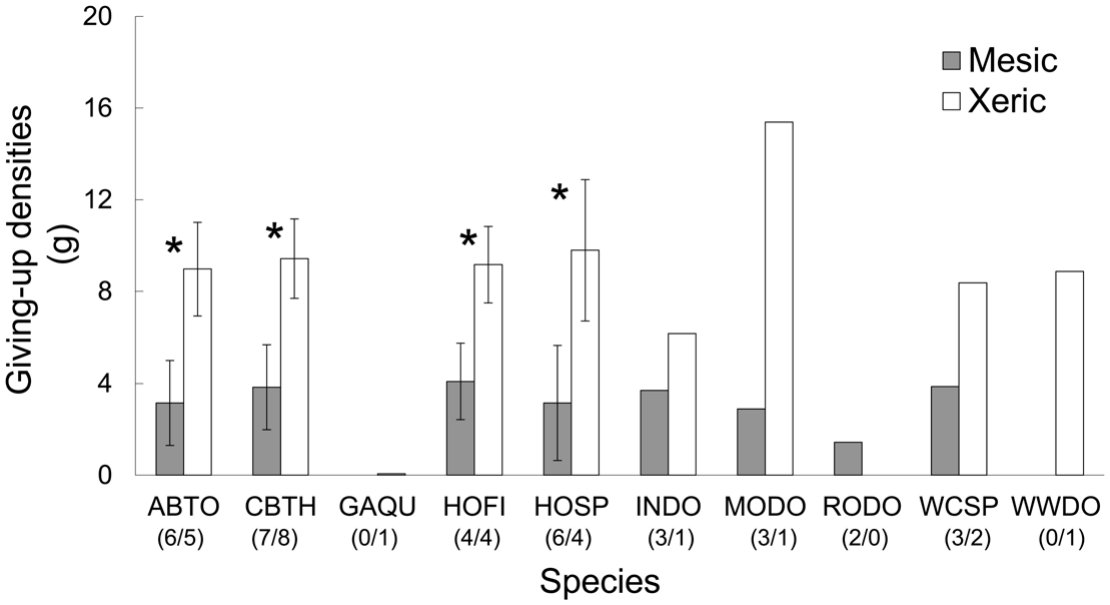
Mean GUD for each species visiting seed trays for mesic and xeric yards. Dark bars represent mesic yards and white bars represent xeric yards. Sample sizes for each yard type shown below species codes (mesic/xeric), standard error bars shown for four most common species (as indicated with asterisk). Species codes are as follows: ABTO = Abert’s towhee, CBTH = curve-billed Thrasher, GAQU = Gambel’s quail, HOFI = house finch, HOSP = house sparrow, INDO = Inca dove, MODO = mourning dove, RODO = rock pigeon, WCSP = white-crowned sparrow, WWDO = white-winged dove. Dark-eyed junco, northern mockingbird, orange-crowned warbler, and spotted towhee also visited the trays but GUDs were not calculated for these species.

Fourteen bird species visited the seed trays. Eleven species were recorded in mesic yards and ten species were recorded in xeric yards (Fig. 2). The majority of species were recorded in both yard types with the exception of rock pigeon (*Columba livia*), dark-eyed junco (*Junco hyemalis*), northern mockingbird (*Mimus polyglottos*) orange-crowned warbler (*Vermivora celata*) (only in mesic yards); white-winged dove (*Zenaida asiatica*), Gambel’s quail (*Callipepla gambelii*) and spotted towhee (*Pipilo maculates*) (only in xeric yards). Curve-billed thrasher, Abert’s towhee, house sparrow, and house finch were the most widespread species (15, 11, 10, and 8 yards respectively). The GUDs for dark-eyed junco, northern mockingbird, orange-crowned warbler, and spotted towhee were not estimated since these birds were never the final forager and therefore we did not have an initial GUD-peck calculation to enter into the model. The bird community did not differ significantly between the two yard types (MRPP, Chance corrected within-group agreement A = 0.002013, P = 0.41).

**Figure 3:**
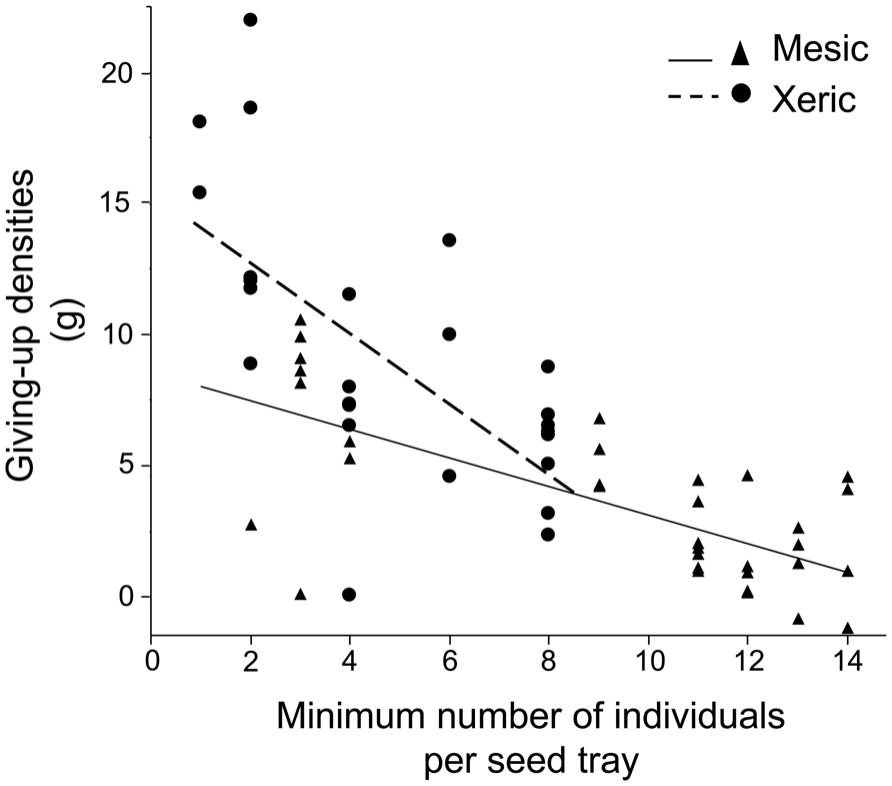
GUDs, bird densities and yard type. The interaction between bird density (minimum individuals visiting a tray) and yard type (mesic: un-dashed line, filled triangles; xeric: dashed line, filled squares) on the GUD. There was a strong interaction between bird density and yard type (ANCOVA, t Ratio = 5.03, *P*<0.0001).

**Figure 4:**
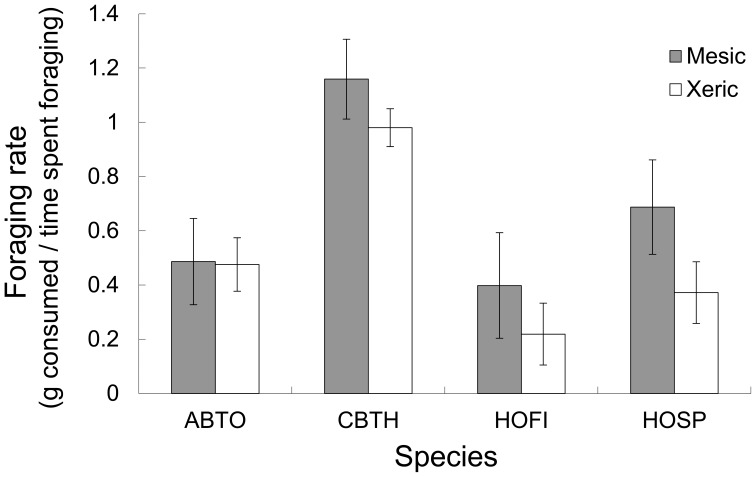
Foraging rates in mesic and xeric yards. Comparison of foraging rate for the four most common species visiting the seed trays for mesic and xeric yards. Dark bars represent mesic yards and white bars represent xeric yards. Species codes are as follows: ABTO = Abert’s towhee, CBTH = curve-billed Thrasher, HOFI = house finch and HOSP = house sparrow. Curve-billed thrasher had the fastest seed consumption rate for both yard types.

We found no evidence that distance from vegetation influenced foraging decisions for either yard type (Paired t-test; mesic yards: t = −0.40, df = 25, P = 0.69; xeric yards: t = 0.56, df = 18, P = 0.58). Therefore we pooled data from the bush and open trays and calculated the mean GUD per species per yard for the remaining analyses. Birds foraging in mesic yards consumed more seed (i.e. lower GUDs) than birds foraging in xeric yards (ANOVA, F = 26.07, df = 2, 59, P<0.0001; mean mesic: 3.39, ±0.57 SE, xeric mean: 9.02±1.1 SE). When we accounted for bird density (i.e. minimum number of individuals visiting a tray) as a covariate on the GUD, the interaction of yard type and bird density was significant (ANCOVA, t = 2.99, P = 0.004, Fig. 3). Although the majority of species that foraged in both yard types showed a trend of consuming less seed (higher GUDs) in xeric yards, we only tested for differences for Abert’s towhee, curve-billed thrasher, house finch, and house sparrow. All four species had higher GUDs in xeric yards (Wilcoxon Test, Z = 1.60, P = 0.11, Z = −2.03, P = 0.04, Z = 1.59, P = 0.11, and Z = 1.79, P = 0.07, respectively), though only curve-billed thrasher was significant at the 0.05 level. These four species also exhibited different foraging efficiency rates (i.e. the rate of grams of seed consumed within 24 h) from one another and these differences were significant in each yard design (Kruskal-Wallis Test, chi square = 10.06, P = 0.02, chi square = 14.04, P = 0.003, mesic and xeric respectively, Fig. 4).

## Discussion

### Yard Design

Foraging decisions differed between mesic and xeric yards whereby birds foraging in mesic yards consumed more seed and quit artificial food patches later than birds foraging in xeric yards. These differences in GUDs from artificial food patches provide important insights into how the birds perceive their environment [33], and reflect the quality of habitat and available resources [34]. Thus, the higher GUDs from the xeric patches suggested that alternative resources might be more abundant there than in mesic yards [19], alternatively, foraging costs were higher in mesic yards. Additional contributing factors for the GUD differences between xeric and mesic yards might include a greater number of individuals foraging in mesic yards (bird densities), differences in species composition between the two yards, or differences in foraging behavior among species. We address each of these possible mechanisms separately.

The most striking difference between the mesic and xeric yards is the presence of turf in mesic yards. The lawn conditions are ideal for granivorous birds [35], and might explain why this guild flourishes in urban environments in general [4] and mesic yards, specifically. For example, Inca dove (*Columbina inca*), rock pigeon, house sparrow, and mourning dove (*Zenaida macroura*) were more prevalent in mesic yards compared with xeric yards (Fig. 2). The success of Inca doves, obligate granivores, in urban yards, and particularly mesic designs, is largely attributable to moist conditions from lawns [36].

### Bird Densities

Regarding the interaction between bird density and GUD (Fig. 3), it appeared that in xeric yards, the number of individuals foraging on a tray drove the GUD patterns; trays with more individual foragers had less seed remaining (i.e. lower GUD) after 24 h. This pattern was similar, though not nearly as strong, in mesic yards. Xeric trays had lower bird densities (minimum of eight individuals) compared with mesic trays (minimum of fourteen individuals). When bird densities for both yard types reached eight individuals (the maximum for xeric yards), the slopes intersected (Fig. 3). Our data suggests that at low densities, birds may not reach their GUD, especially birds foraging in xeric yards. Alternatively, birds might aggregate at sites with low foraging costs, and hence, these patches will have low GUDs.

The GUD method assesses foraging behavior of individuals at the species level and assumes that differences in GUD reflect a species’ foraging traits, in addition to the number of individuals visiting a seed tray (e.g. more individuals might lead to increased competition; [11]). Therefore we suggest that trays with low GUDs can be accounted for by the presence of a particular species able to find additional resources after other species quit foraging the artificial patch. Furthermore, in our study, bird density was strongly correlated with species richness, a relationship expected by chance [5], [37]. Therefore, trays with more individuals also had a greater probability of including a species able to deplete the trays to a lower GUD. Trays in xeric yards with high GUDs and fewer than three individuals (upper left, Fig. 3), were primarily visited by Abert’s towhees and curve-billed thrashers. These species were also present on trays in xeric yards with low GUDs, but the low GUD trays also included house sparrows. Therefore, the lower GUDs from the trays with higher bird densities were likely attributable to this extremely abundant urban species. Furthermore, there were trays from mesic yards with fewer than three individuals and extremely low GUDs, similar to GUDs from trays with more than eight individuals (lower left, Fig. 3). These GUDs were associated with rock pigeons.

### Species Composition and Foraging Behavior

Based on previous studies, we expected the bird composition to differ between the two yard types, with more generalist and invasive species visiting the trays in mesic yards and more native species visiting the trays in xeric yards [6]–[8]. Although the species composition visiting the seed trays were similar between the yard types, a post hoc analysis found that xeric yards exhibited a more even bird community (i.e. individuals were equally abundant among the species) than the mesic yards (ANOVA, F = 5.63, df = 2, 18, P = 0.03). Our results paralleled differences in evenness found when comparing desert and urban bird communities [5]. Nonetheless, the overall similarity in the bird compositions of the two yard types offered the opportunity to test whether individual species shifted foraging behavior between mesic and xeric yards.

The four most abundant species visiting the trays, Abert’s towhee, curve-billed thrasher, house finch, and house sparrow, altered foraging behaviors between the mesic and xeric yards; they all consumed more seed in mesic yards (Fig. 2). The Abert’s towhee, native to the southwestern United States, is typically found along desert streams. Perhaps its capacity to consume more seed in mesic yards derives from habitat similarities between these yards and riparian areas [38], [39]. Fokidis et al. [40] also found behavioral differences between urban and desert Abert’s towhees and curve-billed thrashers in Phoenix, AZ; urban birds were more aggressive than desert birds. In a meta-analysis of bird invasions, Sol and colleagues [41] tested whether behavioral flexibility aided in the success of an invasion. They found that species with larger brains and innovative foraging behavior were more successful invaders of novel environments. The idea that an individual species alters behavior between two different environments suggests the ability to respond to different foraging costs associated with the environments. Furthermore, this ability might explain why these species can acclimate to novel habitats (like the mesic yards). The results also suggest that species unable to alter foraging behavior might be less adept at obtaining resources in novel habitats.

GUDs change with varying risks associated with a species’ environment. Thus, in environments with lower costs, species are expected to be more efficient foragers. Although all species had lower GUDs in mesic yards compared with xeric yards, we did not find GUD differences between species. However, when we calculated the rate of consumption, we found that the curve-billed thrasher consumed seed at a faster rate in both yards compared to its competitors. To explain this conundrum, we suggest that differences in harvesting rates might explain how species coexist in the two yard designs (e.g., [42]). When resources are more abundant, a forager experiences a faster seed encounter rate. Therefore, it might be more advantageous to have a faster foraging rate in rich patches [42]. Although resources were abundant in xeric yards (as indicated by higher GUDs compared with mesic yards), Abert’s towhee, house finch, and house sparrow were not able to consume resources as quickly as the curve-billed thrasher.

We recognize the difficulty of disentangling the effects of species differences and bird densities on the GUD. Both of these factors influence foraging behavior, and our study reveals some possible mechanisms accounting for differences in GUDs for the two yard types: that individual species have diverse foraging strategies, some species can alter foraging strategies, and these strategies enable species to exploit different habitats that vary in habitat quality and available resources.

### Predation

We found no evidence that birds perceived the bush tray as more risky than the open tray, or vice versa. The lack of a difference in foraging decisions between the two trays might also suggest that both trays were equally risky and the birds responded accordingly. However, the risk was not so great that it deterred the birds from foraging and therefore the data suggest the former. The lack of a perceived predation risk for either yard type supports the growing evidence of relaxed predation on adult birds and mammals in cities, regardless of landscape design [10], [15], [43]. As a caveat, we acknowledge that cats kill millions of birds each year [44]. However, our study suggests that perhaps certain bird species might not perceive cats or aerial predators as a threat. Alternatively, species that did not visit the seed trays might be more threatened by cats and other urban predators, and cat presence might explain the absence of some bird species.

### Making GUD Better

Our newly developed analytical approach allowed us to address how foraging decisions might be influenced by all species visiting the seed trays, and not just the final forager as in previous studies [10], [45]. We thus achieved greater power in our interpretation. The information regarding when each individual species quits foraging on a seed tray, relative to the other foragers, suggests how certain species might have a greater influence on the GUD. With the video monitoring, we could accurately identify all species visiting the trays, the proportion of seed each species consumed, and the identity of the final forager. With this information, we gained a better understanding of how bird densities and species identity interacted with the GUD. In a recent study, Kotler et al. [46] attached passive integrated transponder (PIT) tags to gerbils foraging at artificial food patches within an enclosed environment. They were able to calculate time spent foraging for approximately 20% of the food patches. Although this method greatly advances the GUD method, it is highly intensive and when used in wild populations, cannot account for every individual forager. Our video monitoring coupled with our GUD-Peck model can be easily replicated in many field conditions.

Our study is one of the first to use a mechanistic approach to assess the effectiveness of particular residential landscape designs in supporting native bird communities. The xeric yard types had a more even bird community and the higher GUDs were indicative of a superior habitat compared with the mesic yard types. Behavioral indicators such as foraging efficiency aid in conservation measures by alerting land managers to high quality habitats for native species [47], [48]. Our study lends further support for designing urban areas that mimic the vegetative composition and configuration of the wildlands being replaced to help combat the loss of urban biodiversity.
